# Recombinant Hyaluronic Acid Produced in *Escherichia coli*: Purification, Molecular-Size Profile and In Vitro Cellular Performance

**DOI:** 10.3390/ijms27219997

**Published:** 2026-11-08

**Authors:** Giorgia Natalia Iaconisi, Manuela Murano, Nunzia Gallo, Giuseppe Fiermonte, Andrew W. Truman, Natalia M. Vior, Abbas Maqbool, Roberta Del Sole, Yuan Li, Vincenza Dolce, Loredana Capobianco

**Affiliations:** 1 Department of Biological and Environmental Sciences and Technologies, University of Salento, 73100 Lecce, Italy; giorgia.iaconisi@uniba.it; 2 Department of Bioscience, Biotechnology and Environment, University of Bari, 70125 Bari, Italy; giuseppe.fiermonte@uniba.it; 3 Department of Pharmacy, Health, and Nutritional Sciences, University of Calabria, 87036 Cosenza, Italy; manuela.murano@unical.it; 4 Department of Engineering for Innovation, University of Salento, Via Monteroni, 73100 Lecce, Italy; nunzia.gallo@unisalento.it (N.G.); roberta.delsole@unisalento.it (R.D.S.); 5 Department of Molecular Microbiology, John Innes Centre, Colney Lane, Norwich NR4 7UH, UK; andrew.truman@jic.ac.uk (A.W.T.); natalia.miguel-vior@jic.ac.uk (N.M.V.); 6 Department of Biophysical Analysis Platform, John Innes Centre, Norwich NR4 7UH, UK; abbas.maqbool@jic.ac.uk; 7 School of Pharmaceutical Sciences, Institute of Materia Medica, Xinjiang University, Urumqi 830017, China; liyuanstc@xju.edu.cn

**Keywords:** hyaluronic acid, recombinant production, purification, molecular-size distribution, mass photometry, rheology, cytocompatibility

## Abstract

Recombinant production platforms are promising approaches for producing hyaluronic acid (HA) for biomedical research. In this study recombinant HA produced in *Escherichia coli* (rHA) was purified and compared with commercial HA (cHA) through physicochemical, rheological, and preliminary cellular analyses. Purification reduced residual protein content (from 7.47 to 0.48 mg/L), nucleic-acid-associated absorbance and endotoxins level (from 0.315 to 0.0766 EU/mg) while preserving HA recovery. Electrophoresis and hyaluronidase digestion supported HA identity and a heterogeneous apparent molecular-size profile. Mass photometry provided an exploratory, solution-based estimate of molecular-size populations, identifying a predominant native-solution population centred at approximately 194 kDa. Fourier-transform infrared spectroscopy (FT-IR) revealed characteristic HA-associated functional groups, while thermogravimetric analysis (TGA) showed an initial mass loss attributed to water evaporation (30–150 °C), followed by polymer degradation occurring at a lower temperature for rHA (~150 °C) than for cHA (~250 °C). Both samples exhibited shear-thinning behaviour, whereas rHA showed lower viscosity and moduli than cHA. In C2C12 cells, rHA produced favourable relative sulforhodamine B (SRB) signals and scratch closure responses comparable to those of the two cHA (cHA_1 and cHA_2). These findings support rHA as a purified HA-compatible material with distinctive rheological properties and preliminary in vitro cellular performance.

## 1. Introduction

Hyaluronic acid (HA), also known as hyaluronan, plays a pivotal role in biomedical applications due to its unique physicochemical and biological properties. HA is a member of the glycosaminoglycan (GAG) family, distinguished by its high degree of polymerization and distinctive macromolecular structure. It is widely distributed in connective, epithelial, and neural tissues, where it contributes to hydration, viscoelasticity, and the structural integrity of the extracellular matrix [1,2,3].

Unlike other GAGs such as chondroitin sulphate, dermatan sulphate, heparan sulphate, and keratan sulphate, HA is not sulphated along its polymer chain. It is synthesized on the cytoplasmic surface of the plasma membrane by hyaluronan synthase (HAS) enzymes [4], rather than in the rough endoplasmic reticulum and Golgi apparatus. Another distinctive feature of HA is that it does not attach to a core protein, which sets it apart from other GAGs [1,5]. Structurally, HA is composed of repeating disaccharide units of β-(1,4)-glucuronic acid (GA) and β-(1,3)-N-acetylglucosamine (NAG), connected alternately by β-1,3 and β-1,4 glycosidic bonds [5]. The presence of carboxylic groups in HA makes it negatively charged and highly hydrophilic at physiological pH. This property enables HA to bind substantial amounts of water, creating a viscous structure through intramolecular and intermolecular hydrogen bonding [6,7].

HA plays a major role in maintaining extracellular matrix hydration, where it acts as a water-binding component [8]. This function enhances tissue resistance to physical stress and ensures adequate moisture levels. Additionally, HA contributes to tissue homeostasis and supports the growth of epithelial cells, eosinophils, and macrophages. It forms a pericellular layer around several cell types, where its molecular weight influences its role as a signalling molecule and its regulation of cell adhesion, migration, and proliferation [9].

HA can be classified into three categories based on its molecular weight: (i) low-molecular-weight (LMW) HA (<200 kDa); (ii) medium-molecular-weight (MMW) HA (0.2–1.0 MDa); (iii) and high-molecular-weight (HMW) HA (>1 MDa) [1,10]. The polymer can consist of up to 10,000 or more disaccharide units, yielding a molecular mass of approximately 4 million Da [5,11]. The molecular weight of HA is closely linked to its biological activity. The size of the molecule affects its receptor binding affinity, ability to form receptor complexes, and cellular absorption [12]. As the molecular weight of HA increases, so does its mechanical strength. Thus, HMW HA is preferred in applications requiring strong viscoelastic and mechanical properties, such as joint supplements and wound dressings [13]. HMW HA is also noted for its anti-inflammatory and immunosuppressive properties and its involvement in angiogenesis, making it suitable as a space filler and a natural immunologic depressant [5]. HMW HA is a crucial component of synovial fluid, where it serves as a vital lubricant for joints [9]. This function makes it significant in both normal physiological processes and pathological conditions, such as osteoarthritis [14]. Conversely, MMW and LMW HA exhibit different properties. These forms are known to stimulate the synthesis of heat-shock proteins, providing proangiogenic, antiapoptotic, and immunostimulatory effects [5,15]. LMW HA, in particular, has found widespread use in cosmetic and dermatological applications due to its ability to penetrate the epidermal layer and enhance tissue hydration and healing [16].

Its high biocompatibility, biodegradability, and bioactivity make HA an ideal material for regenerative medicine, tissue engineering, drug delivery, and aesthetic medicine [1,5]. More broadly, the development of advanced polymeric materials for healthcare applications increasingly aims to combine physicochemical performance with additional functional properties, including antibacterial activity, as reported for Cu- and Ag-based treatments applied to polymeric substrates [17]. Indeed, HA-based formulations are successfully applied in osteoarthritis treatment, chronic wound healing, and ophthalmic surgery, confirming its versatility and central role as one of the most promising biomaterials in modern biomedical science [18].

Owing to its remarkable bioactivity, HA has attracted increasing attention as a biomaterial for the development of healthcare products [19,20]. The growing demand for HA in regenerative medicine, ophthalmology, osteoarthritis treatment, aesthetic medicine, and advanced drug delivery systems has highlighted the need for scalable, reproducible, and high-purity production processes [21]. Conventional extraction methods from animal tissues (e.g., rooster combs, bovine vitreous humor, or umbilical cords) remain a viable source of HA [22,23]. However, these traditional approaches are associated with several limitations, including low and variable yields, broad molecular weight distributions, extensive use of organic solvents and enzymatic treatments, difficulty in separating HA from structurally similar glycosaminoglycans and proteoglycan complexes, and an increased risk of contamination with host proteins, endotoxins, nucleic acids, and potential pathogens [24]. Collectively, these factors result in complex and costly purification procedures, poor batch-to-batch consistency, and safety concerns that limit scalability and suitability for high-purity applications [25]. To overcome these limitations, microbial fermentation has become the preferred industrial route for HA production. Strains such as *Streptococcus zooepidemicus* have been traditionally employed due to their natural ability to synthesize HA as a capsular polysaccharide [26].

Microbial fermentation of *S. zooepidemicus* has been extensively investigated at both laboratory and industrial scales, with HA production strongly depending on the strain and fermentation conditions [5,27]. In particular, fermentation of *Streptococcus equi* subsp. *equi* under optimized conditions yielded approximately 12 g/L HA [28]. At bioreactor scale, *Streptococcus zooepidemicus* fermentation has also been reported to produce approximately 5.2–5.8 g/L HA, while a downstream recovery process based on acidification, activated-carbon treatment, ultrafiltration/diafiltration, and isopropanol precipitation achieved 76% recovery and 99.6% purity [29]. These results highlight that, in addition to fermentation productivity, downstream processing is a critical determinant of the recovery and purity of HA intended for biomedical applications [30].

More recently, recombinant *Bacillus subtilis* or *Escherichia coli* have been exploited to enhance yield, control molecular weight, and eliminate the risk of pathogen-derived impurities [31,32,33,34]. In a comparative study of recombinant bacterial hosts, Nasser et al. reported HA titres ranging from 5–585 mg/L in different *E. coli* strains and up to approximately 2.5 g/L in *Bacillus megaterium*, demonstrating the strong influence of the host, HA biosynthetic gene set, and culture conditions on both production yield and polymer molecular characteristics [31].

More broadly, the development of recombinant production platforms benefits from advances in genome-engineering strategies designed to promote stable and predictable transgene expression, including the identification and characterization of genomic safe-harbor sites [35]. In addition, advancements in enzymatic and cell-free biosynthetic approaches offer new perspectives for highly controlled, animal-free, pathogen-free, and sustainable HA synthesis, though they remain limited by production cost and scalability [36].

In this context, this work aimed to investigate the structural and functional properties of a recombinant HA (rHA) produced by *E. coli* according to a proprietary process in comparison with a commercial analogue (cHA_1). Particular emphasis was placed on purity assessment, electrophoretic and mass photometric evaluation of the apparent molecular-size distribution, light scattering, spectroscopic characterization, thermal behaviour, and rheological properties. In addition, preliminary in vitro assays were performed to evaluate biocompatibility and the ability of rHA to support cellular responses relevant to tissue repair.

## 2. Results

### 2.1. rHA Purification

rHA was produced in E. coli using a proprietary and optimized fermentation process. Briefly, the bacterial host was genetically engineered to express the genes involved in HA biosynthesis, followed by induction and optimization of their expression under controlled fermentation conditions. Production was carried out in a nutrient-rich medium, with key fermentation parameters monitored and controlled to support bacterial growth and HA production. Due to ongoing intellectual property protection, detailed information cannot be disclosed [r. o. Italian patent N. 102025000010455]. Following microbial production, rHA was recovered and subjected to downstream processing steps designed to remove cellular components and other impurities.

**Figure 1:**
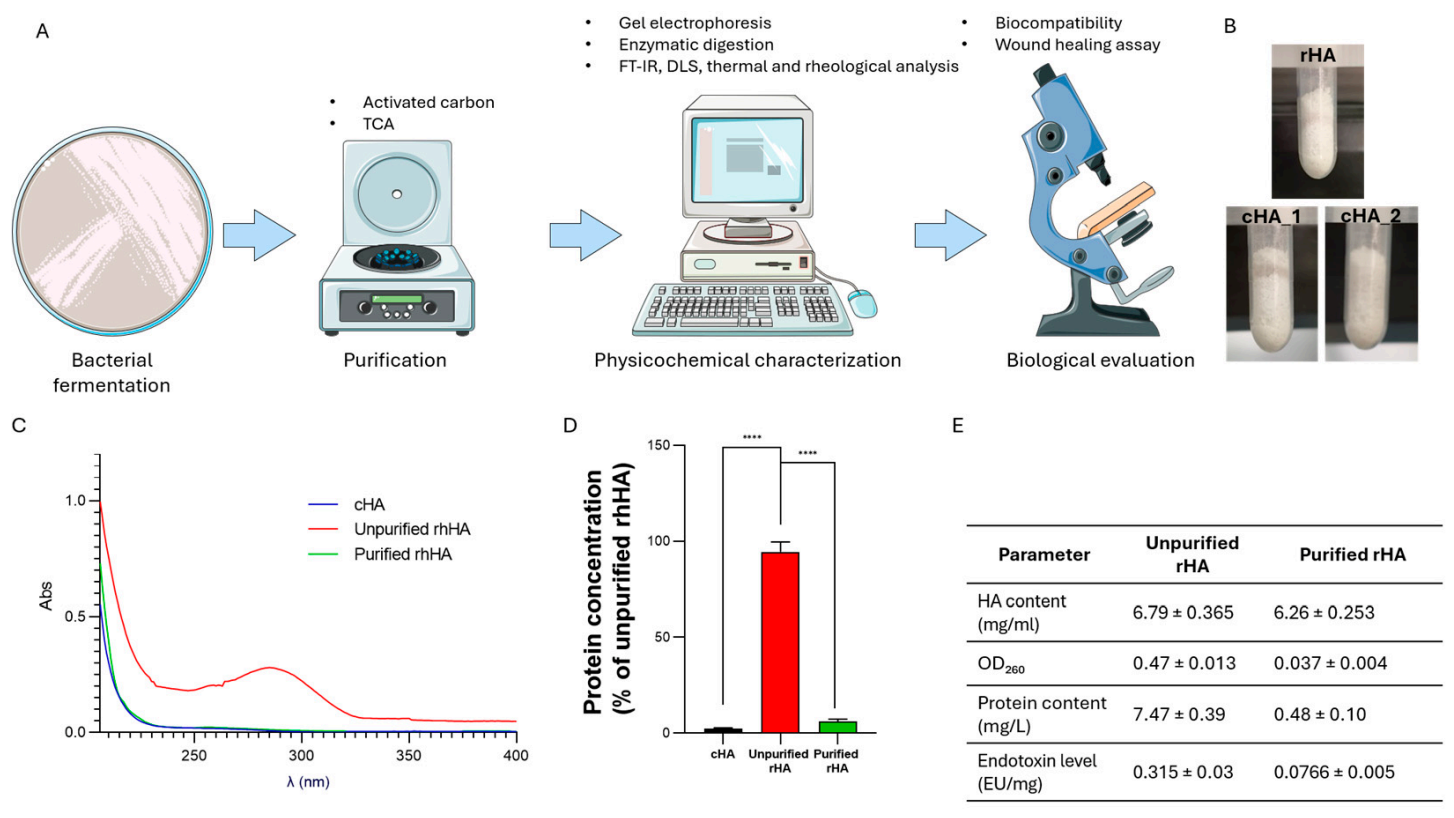
Production workflow and purity assessment of rHA. (**A**) Workflow of rHA evaluation and characterization. The workflow illustrates rHA recovery from fermentation, followed by purification steps (activated carbon, TCA treatment), physicochemical characterization (UV-Vis, FT-IR, gel electrophoresis, TGA, rheology), and biological evaluation using C2C12 cells for biocompatibility and wound healing assays. (**B**) Photograph of freeze-dried purified rHA obtained after the purification process, cHA_1 and cHA_2. (**C**) UV-Vis spectra of purified (green line) and unpurified (red line) rHA in comparison with cHA_1 (blue line). (**D**) Protein content of HA samples determined by Bradford assay and represented as a bar chart. Statistical significance between groups is indicated as **** p < 0.0001 (**E**) Summary of purification efficiency showing HA recovery, protein content, A260 values and endotoxin level before and after purification. Adapted from Servier Medical Art “https://smart.servier.com (accessed on 27 July 2026)”, licensed under CC BY 4.0 “https://creativecommons.org/licenses/by/4.0/ accessed on 27 July 2026)”.

The overall process generated a product suitable for subsequent physicochemical and biological characterization (Figure 1, panel A), while preserving the structural integrity of the polymer.

The effectiveness of the purification process was evaluated by determining HA recovery, protein content, and nucleic-acid-associated absorbance (Figure 1, panels D,E). The HA concentration was only marginally affected by purification, decreasing from 6.79 ± 0.365 mg/mL to 6.26 ± 0.253 mg/mL (*p* < 0.05), indicating that the downstream process preserved most of the polymer while removing contaminants (Figure 1, panel E). Similar observations have been reported for HA purification procedures based on adsorption and precipitation strategies, which efficiently remove impurities while maintaining high HA recovery yields [29].

UV–Vis spectroscopic analysis showed that purified rHA exhibited a spectral profile comparable to that of commercial HA (Figure 1, panel C). An absorption feature at approximately 280 nm was detected only in unpurified rHA, consistent with the presence of residual proteins and other fermentation-derived contaminants. The disappearance of this signal after purification indicated effective contaminant removal.

Moreover, unpurified rHA showed higher absorbance at 260 nm (A260 = 0.47 ± 0.013), consistent with a greater content of nucleic-acid-associated and other UV-absorbing fermentation-derived impurities. The purification process markedly reduced this value to A_260_ = 0.037 ± 0.004, reaching a level comparable to that of commercial HA (A_260_ = 0.032 ± 0.002) (Figure 1, panels D,E).

Notably, protein levels in purified rHA were markedly reduced, although they remained higher than those measured in cHA_1 (cHA_1 protein content: 0.19 ± 0.009 mg/L; A260 = 0.032 ± 0.002), indicating the effective removal of fermentation-derived impurities.

Endotoxin levels were also reduced following purification, decreasing from 0.315 ± 0.03 to 0.0766 ± 0.005 EU/mg (Figure 1, panel E).

### 2.2. HA Molecular Mass Distribution

The apparent molecular mass distribution of rHA was investigated by gel electrophoresis followed by densitometric analysis. Polyacrylamide gels were employed as the primary method, as this matrix effectively separates HA in the 5–500 kDa range and provides excellent staining clarity [37].

**Figure 2:**
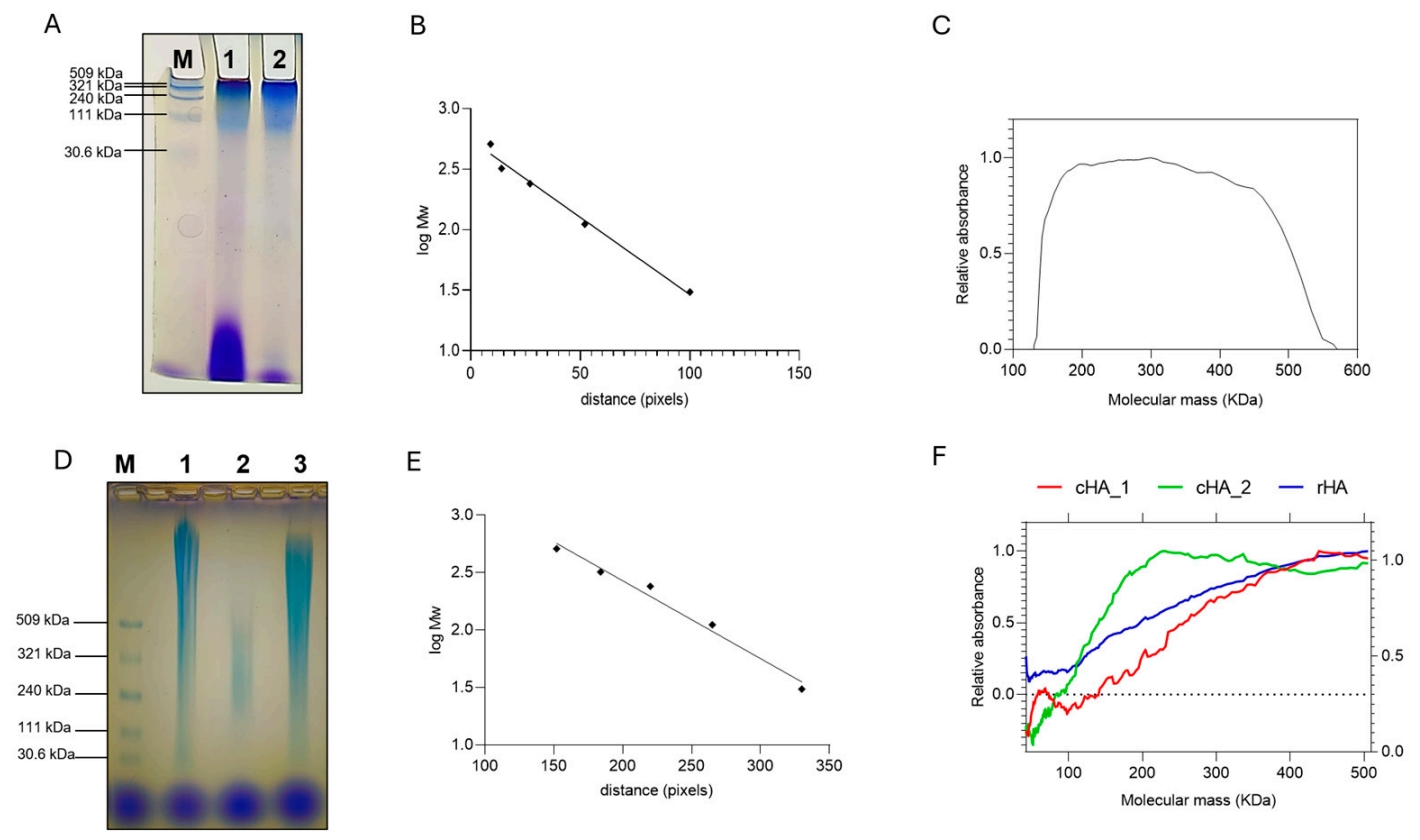
Electrophoretic and densitometric characterization of rHA. (**A**) Polyacrylamide gel electrophoresis (4–20%, TBE buffer) of cHA and rHA samples. Lane M: HA molecular weight standards; Lane 1: pre-purification rHA; Lane 2: purified rHA. The pre-purification sample displayed broad electrophoretic smearing predominantly in the lower region, whereas the purified sample showed more defined migration with reduced smearing. (**B**) Calibration curve generated using HA molecular weight standards (509, 321, 240, 111, and 30.6 kDa) electrophoresed on the same PAGE gel. Linear regression analysis: log M = −0.01215 × pixel position + 2.752, R^2^ = 0.9859. (**C**) Normalized densitometric profile of purified rHA derived from PAGE, showing the apparent molecular mass distribution. The profile reaches its maximum at approximately 300 kDa, with a plateau between approximately 170 and 400 kDa, within the calibrated interval. (**D**) Agarose gel electrophoresis (2% agarose, TBE buffer). Lane M: HA molecular mass standards; lane 1: cHA_1; lane 2: cHA_2; lane 3: purified rHA. (**E**) Calibration curve generated using HA standards for agarose gel; linear regression log M= −0.0068 × pixel position + 3.7846, R^2^ =0.9805. (**F**) Background-corrected densitometric profiles of cHA_1, cHA_2, and purified rHA. Each profile was normalized to its own maximum staining intensity. The conversion of migration distance into apparent molecular mass is restricted to the calibrated range.

As shown in Figure 2, panel A the pre-purification rHA sample (lane 1) displayed a broad electrophoretic smear extending toward the lower region of the gel, consistent with a heterogeneous population enriched in lower-molecular-weight HA species and degradation fragments. In contrast, the purified rHA sample (Figure 2, panel A, lane 2) exhibited a more defined migration pattern with markedly reduced smearing. These changes are consistent with an improvement in electrophoretic resolution after purification; however, gel electrophoresis alone does not demonstrate a quantitative enrichment of higher-molecular-mass species. To estimate the apparent molecular mass distribution, a calibration curve was generated using HA molecular weight standards (509, 321, 240, 111, and 30.6 kDa) electrophoresed on the same gel (Figure 2, panel B). Linear regression analysis of log molecular weight versus migration distance (pixel position) revealed a satisfactory correlation (R^2^ = 0.9859) within the linear separation range, which was subsequently used to convert migration profiles into apparent molecular mass distributions. Densitometric analysis of purified rHA (Figure 2, panel C) revealed a broad molecular mass profile characteristic of polydisperse HA populations. The normalized densitometric profile indicated the presence of multiple molecular mass species spanning a wide apparent molecular weight range, with a plateau between approximately 170 and 400 kDa, that is, entirely within the interval bracketed by the HA standards. Preliminary mass photometry analysis (Supplementary Figure S2) further supported the heterogeneous nature of the purified rHA preparation, supporting the polydisperse character suggested by gel electrophoresis.

Since PAGE provides limited resolution for larger HA species, agarose gel electrophoresis was performed as a complementary method. Agarose gels offer improved separation of HA populations in the higher apparent molecular mass range, enabling better visualization of medium- and high-molecular-weight species [38]. To compare the recombinant product with commercial HA preparations, agarose gel electrophoresis was performed (Figure 2, panel D). A calibration curve was generated using the same HA molecular weight standards (Figure 2, panel E). Material migrating more slowly than the 509 kDa standard was considered above the calibrated range.

Apparent molecular masses above the highest standard were therefore removed from the converted profile, which is restricted to the calibrated range (Figure 2, panel F).

Background-corrected densitometric profiles (Figure 2, panel F) revealed notable differences in the apparent molecular mass distribution profiles between cHA_1 (red line), cHA_2 (green line) and purified rHA (blue line), indicating differences in polydispersity and/or fragmentation state between the samples.

Both cHA_1 and purified rHA exhibited broad profiles extending across much of the analyzed migration interval, although their intensity distributions differed. In contrast, cHA_2 showed a more restricted profile, with most of the detectable signal located within the migration region approximately bounded by the 509 and 111 kDa standards. These observations are consistent with differences in the apparent molecular mass heterogeneity of the preparations.

Each profile was normalized to its own maximum intensity to facilitate comparison of profile shape. Consequently, the normalized curves do not provide a quantitative comparison of HA abundance between samples. Differences between the PAGE and agarose profiles should also be interpreted in light of their different separation ranges and resolution, particularly for material outside the calibrated interval.

The identity and specificity of the purified product was further verified by enzymatic digestion using hyaluronidase type V, an enzyme that selectively hydrolyses the β-(1→4) glycosidic bonds of hyaluronic acid (Figure S1). As shown in Figure S1, untreated cHA_1 (Lane 1) and purified rHA (Lane 4) displayed intense blue-stained material with limited electrophoretic migration, with a substantial fraction remaining close to the loading wells. This behaviour is consistent with the low electrophoretic mobility expected for high-molecular-size and/or highly hydrated HA species under the electrophoretic conditions used. Following enzymatic digestion (Figure S1, lanes 2 and 5), this HA-associated material was markedly reduced or disappeared, with the concomitant appearance of faster-migrating low-molecular-weight fragments, consistent with enzymatic degradation of the HA polymer. This electrophoretic shift is consistent with cleavage of the polymer backbone by hyaluronidase and supports the susceptibility of the recombinant product to HA-specific enzymatic degradation. Importantly, the digestion profiles of rHA and cHA_1 were highly comparable, supporting the preservation of HA motifs recognized by hyaluronidase. The substantial reduction in low-mobility polymeric bands after digestion indicates that the major rHA species are susceptible to enzymatic hydrolysis, confirming the HA identity of the recombinant polymer. However, this assay should not be interpreted as absolute exclusion of minor chemically modified, crosslinked, or degradation-resistant fractions that may be present below the detection limit of the gel-based method. Chondroitin sulphate (CS) included as a negative control (Figure S1, lane 7) exhibited a distinct electrophoretic migration pattern and characteristic yellow staining with Stains-All, consistent with the different chemical composition and charge distribution of sulfated glycosaminoglycans.

Indeed, Stains-All is a cationic carbocyanine dye that produces differential coloration of glycosaminoglycans on polyacrylamide gels [38]. Under the staining conditions used here, HA appeared blue, whereas the chondroitin sulphate control appeared yellow.

Importantly, CS digestion with hyaluronidase (Figure S1, lane 8) did not produce electrophoretic changes comparable to those observed for HA samples (Figure S1, lanes 2 and 5), supporting the substrate specificity of the enzymatic treatment and further confirming the identity of the recombinant polymer as HA.

### 2.3. FT-IR Analysis

**Figure 3:**
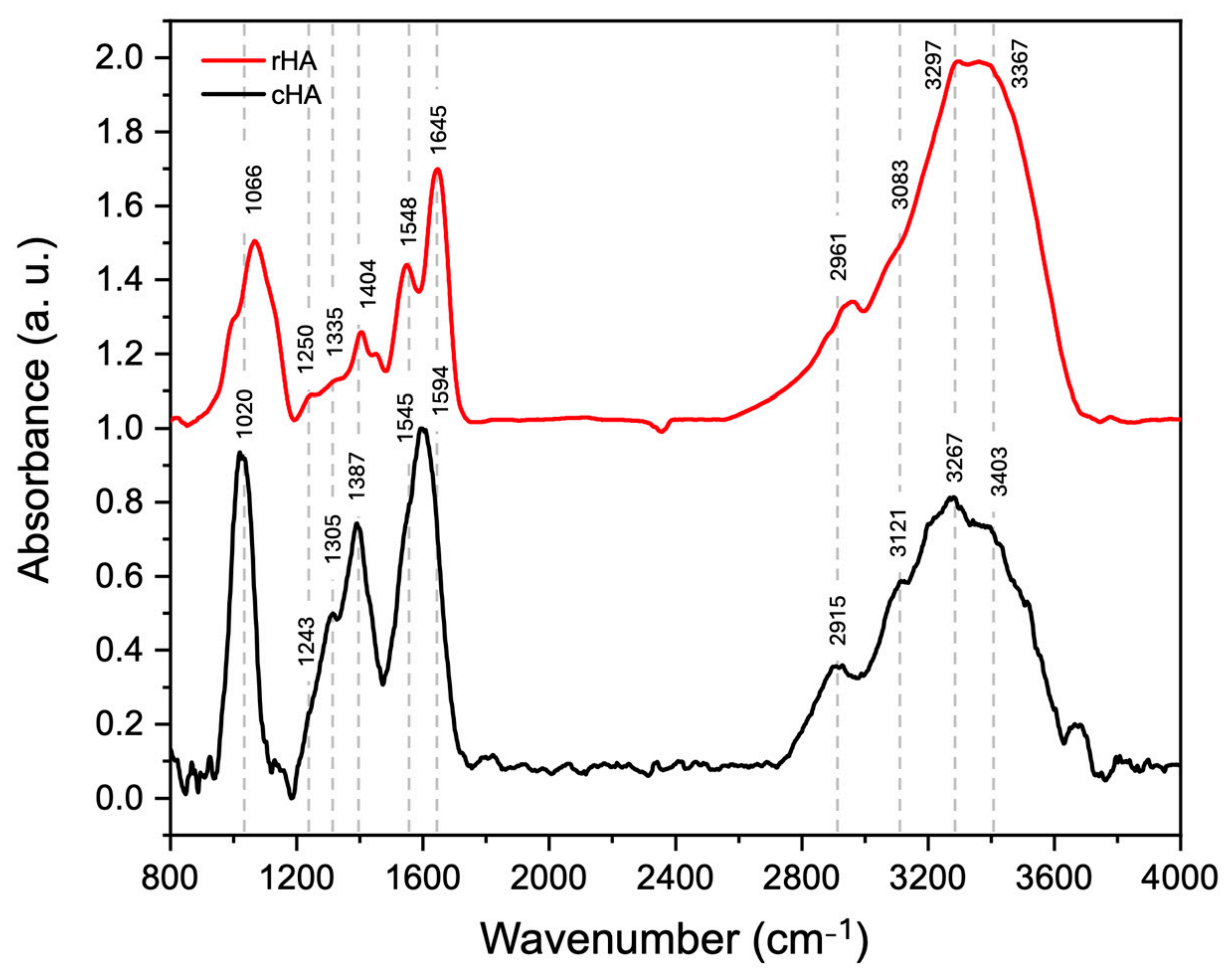
FT-IR spectra of purified rHA and cHA_1. The characteristic absorption bands corresponding to hydroxyl (−OH), amide (C=O, N-H), carboxylate (−COO^−^), and saccharide functional groups are detected, highlighting the structural similarities between the two samples.

FT-IR spectroscopy was used to analyse the purified rHA exposed chemical groups. The cHA_1 sample was also analysed for comparison. The resulting spectra were reported in Figure 3. Both samples exhibited the characteristic absorption bands of key functional groups of HA, confirming its nature by its chemical composition [39,40,41,42,43]. A broad absorption band around 3400 cm^−1^ was found, attributable to the stretching vibrations of the OH and N-H groups that confirmed the strong hydrogen bonding network typical of hyaluronic acid [39,41]. The peaks at approximately 2900 cm^−1^ (2961 cm^−1^ for rHA, 2915 cm^−1^ for cHA) corresponded to the C-H stretching vibrations, further confirming the presence of aliphatic structures within the molecule [40]. In the fingerprint region, the rHA spectrum shows peaks at 1650 cm^−1^ associated with C=O asymmetric stretching of amide I, at 1400 cm^−1^ associated to C–N stretching and N–H bending vibrations associated with amide groups, at 1540 cm^−1^ associated to amide II (N-H bending) vibrations and at 1330 cm^−1^ associated to amide III.

The bands 1411 and 1242 cm^−1^ corresponded to the symmetric and asymmetric stretching vibrations of carboxylate (−COO^−^) moieties, essential groups of the polyanionic HA. According to literature, the shift of those bands is attributable to protonation of the HA molecule due to the use of the trichloroacetic acid [39].

Furthermore, a characteristic band of the alcohol group (C-OH) group was present at about 1050 cm^−1^ (1020 cm^−1^ for cHA, 1066 cm^−1^ for rHA) [41]. A partially hidden peak is present at about 1000 cm^−1^, indicative of the ether (C-O-C) bond [40].

The FT-IR analysis confirms that the rHA exhibits a highly similar spectrum to that of the cHA_1 sample, indicating that the biosynthetic process successfully replicates the natural polymer’s structure. The preservation of key functional groups, including hydroxyl, amide, carboxylate, and saccharide moieties, suggests that the rHA maintains the expected molecular architecture. Despite the overall spectral alignment, minor differences in peak position and intensity may reflect variations in molecular weight and degree of hydration. However, although powerful, FTIR analysis did not allow for an estimate of HA molecular weight. According to the study of Huang et al., this could be due to carbon–oxygen double bond-associated band (1673 cm^−1^) that can overlap with other bands, making it hard to distinguish the molecular size of the HA [44]. These discrepancies could arise from different purification techniques, which may slightly alter hydrogen bonding interactions and overall conformational stability. However, these variations do not compromise the fundamental structure of the rHA, as evidenced by the strong similarity to the commercial counterpart.

### 2.4. Hydrodynamic Size Measurements

**Figure 4:**
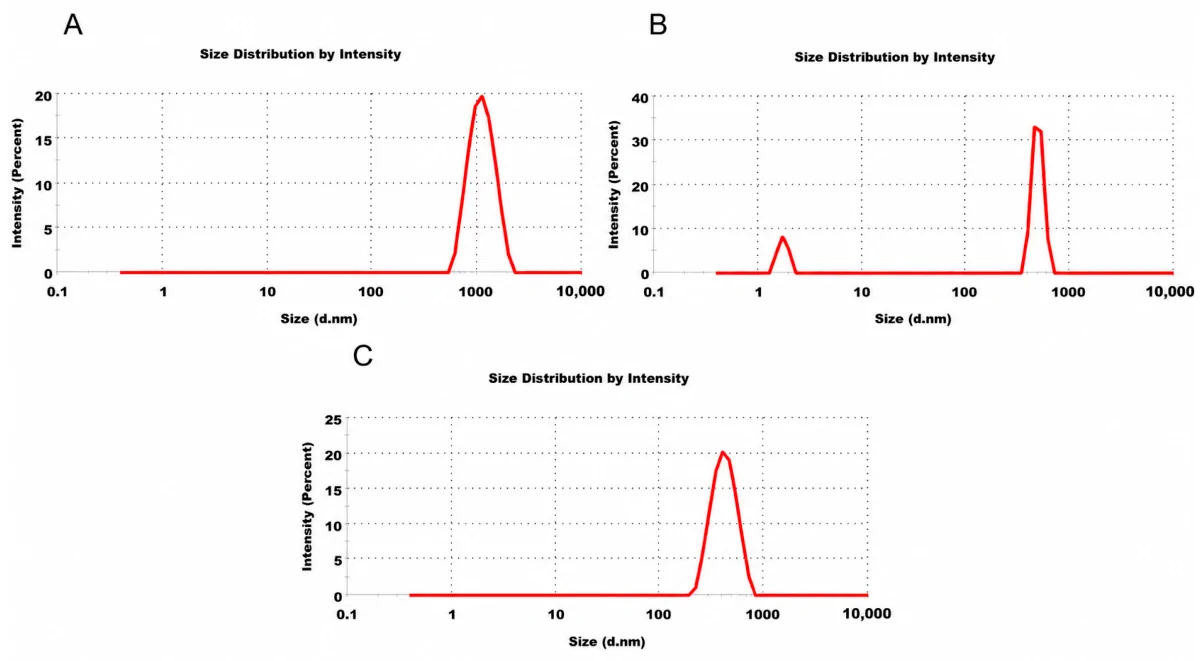
DLS analysis of HA samples. Representative intensity-weighted particle size distributions obtained by DLS for the three analyzed samples: (**A**) cHA_1, (**B**) cHA_2 and (**C**) rHA. Particle size is reported as hydrodynamic diameter (d.nm) on a logarithmic scale, while the y-axis represents the relative scattering intensity (%).

Dynamic light scattering (DLS) analysis was performed on rHA, cHA_1, and cHA_2, and the results are shown in Figure 4. The average hydrodynamic sizes was found to be of 2573 nm with a polydispersity index (PdI) of 0.584 and 1066 nm (PdI = 0.431) for cHA_1 (Sigma) and cHA_2 respectively. The hydrodynamic diameter obtained for rHA was equal to 495 nm (PdI = 0.427). In conclusion, DLS study showed that rHA sample has large average hydrodynamic sizes with PdI values of 0.427. The highest polydispersity and size dimensions were recorded cHA_1, whereas rHA displayed the lowest, yet still highly polydisperse profile. It can be hypothesized that the presence of high polydispersity index can be mostly due to the presence of large particles (large aggregates or visible particulates) or potential sedimentation phenomena that can mask smaller particles making the reported size poorly representative of the primary population.

However, the interpretation of DLS measurements for polymeric systems requires particular caution. DLS determines the hydrodynamic diameter assuming materials particles spherical geometry [45]. This approximation may not accurately describe the actual dimensions of polymers which can adopt non-spherical conformations in aqueous solutions. Consequently, the measured hydrodynamic diameter should be regarded as an apparent sphere-like value rather than a direct measurement of individual polymer chain dimensions. Furthermore, the relatively high PdI values observed for all samples could potentially arise from differences in polymer chain conformations, intermolecular associations, and aggregation phenomena. In addition, the hydrodynamic behavior of HA chains may be influenced by experimental conditions, including polymer concentration, ionic strength, and pH.

Therefore, the DLS results should be interpreted as a comparative assessment of the apparent hydrodynamic behavior of the investigated HA samples under the experimental conditions rather than as an accurate determination of individual polymer chain dimensions. Complementary characterization techniques would be required to provide a more comprehensive description of polymer chain size, conformation, and aggregation state.

### 2.5. Thermal Analysis

**Figure 5:**
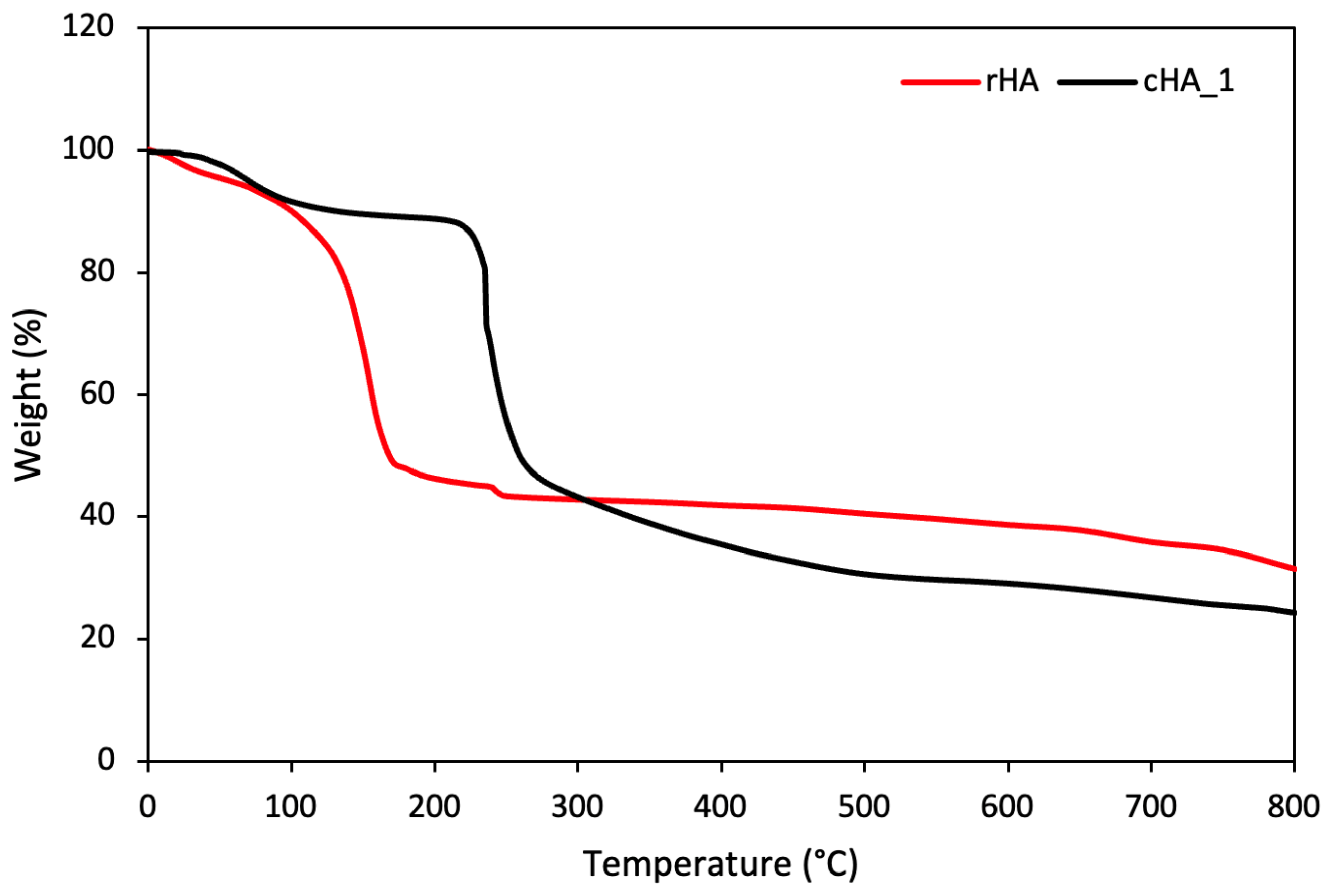
TGA curve of rHA in comparison with cHA_1. The curves represent sample weight loss as a function of temperature. The initial mass loss between approximately 30 and 150 °C is attributed to water evaporation, followed by the main thermal degradation event of the polymers between approximately 150 °C for rHA and 250 °C for cHA_1 and progressive decomposition of the residual material at higher temperatures.

The thermogravimetric analysis (TGA) of rHA in comparison with cHA_1 is illustrated in Figure 5. Although similar, thermograms revealed differences in materials properties. The TGA profile of rHA shows an initial weight loss (~15–20%) between 30 °C and 150 °C, attributed to the evaporation of adsorbed and bound water molecules within the polymer matrix, in agreement with previous TGA studies on recombinant HA [46], while in cHA_1 this phenomenon is extended up to 200 °C.

The main thermal degradation occurs at 150–250 °C with a sharp weight loss, corresponding to the thermal decomposition of the polysaccharide backbone, as previously reported for HA obtained from *S. zooepidemicus* [46]. However, this second phenomenon occurred at lower temperatures for rHA compared to cHA. In particular, rHA showed a pronounced weight loss approximately at 150 °C, while cHA_1 remained comparatively stable up to approximately 250 °C and underwent its major degradation step at higher temperatures.

Above 300 °C, a gradual mass loss is observed for both, associated with the progressive degradation of the remaining organic fractions. The lower rate of weight loss at higher temperatures suggests the formation of a thermally stable residual fraction.

Overall, even if these results indicated a lower apparent thermal stability of rHA compared to cHA_1, it could be assumed that the thermal degradation profile of rHA is consistent with previous reports on natural HA, since it displays the characteristic stages of dehydration, polymer decomposition, and residual mass formation.

### 2.6. Rheological Properties

**Figure 6:**
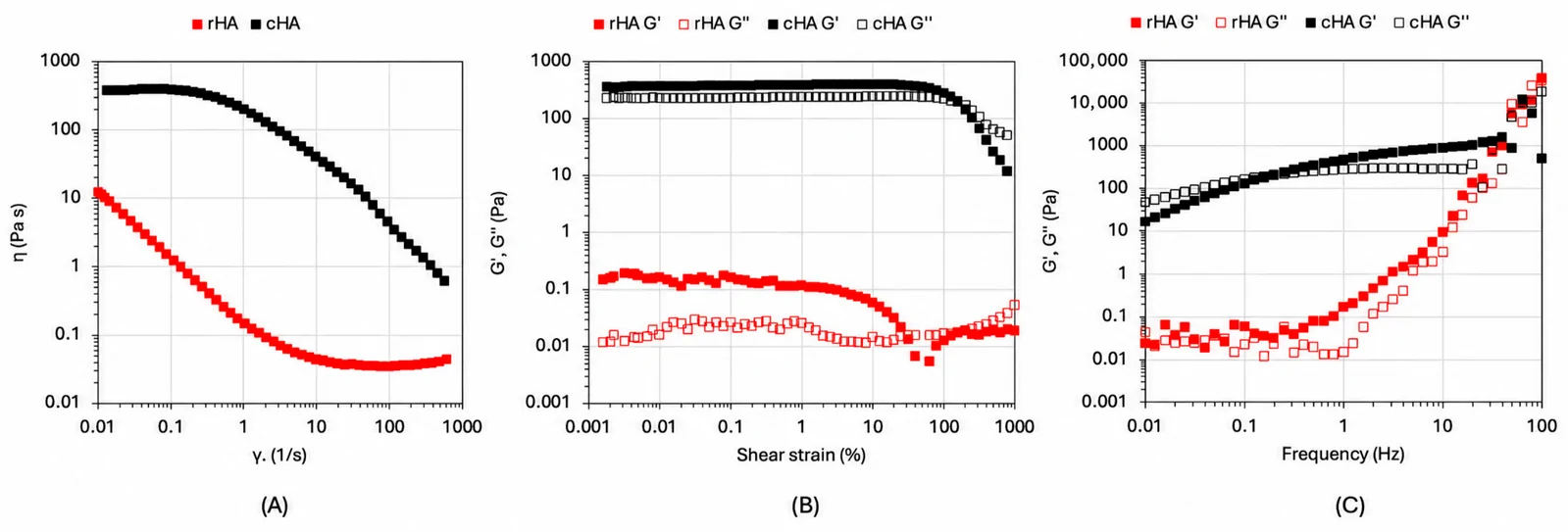
Rheological characterization of rHA in comparison with cHA. (**A**) Shear viscosity measured as a function of shear rate, revealing typical shear-thinning behaviour of HA; (**B**) amplitude sweep test at 1 Hz showing the storage modulus G′ higher than the loss modulus G″, as a function of shear strain; (**C**) frequency sweep at 1% shear strain displaying G′ and G″ increasing with frequency.

HA is a natural polymer that exhibits viscoelastic properties that depend on its molecular weight and concentration. Thus, the viscoelastic properties of rHA were assessed through shear viscosity, amplitude sweep and frequency sweep analyses and are reported in Figure 6. The cHA_1 was also analysed for comparison. Both rHA and cHA_1 exhibited pronounced shear-thinning behaviour (Figure 6, panel A), which is characteristic of hyaluronic acid and indicates non-Newtonian flow properties. As the shear rate increased, viscosity progressively decreased over several orders of magnitude, indicating alignment of polymer chains and progressive disruption of entanglements under flow. The cHA_1 sample showed markedly higher viscosity in the low-shear region, suggesting a more entangled and structured polymer network, potentially associated with differences in molecular-weight distribution and intermolecular interactions. In contrast, rHA exhibited significantly lower viscosity values across the entire shear-rate range. This response is compatible with a lower and broadly polydisperse apparent molecular-mass distribution, as inferred from gel electrophoresis and supported by mass photometry, although rheological data alone cannot be used as an absolute molecular-weight determination.

Amplitude sweep tests were conducted to assess the mechanical stability of the polymer network and to define the linear viscoelastic region (LVR) (Figure 6, panel B). The cHA_1 sample exhibited a wider LVR, with G’ remaining nearly constant up to relatively high strain values (≈70%), indicating high resistance to deformation and robust network integrity. In contrast, rHA shows much lower moduli and a narrower LVR (≈5%), with an earlier deviation from linear viscoelastic behaviour.

Oscillatory frequency sweep tests were performed within the LVR, at a shear strain of 1% (Figure 6, panel C). For cHA, both G′ and G″ moduli were relatively high, with a frequency dependent behaviour. Indeed, G’’ was found to exceed G’ at low frequencies, index of a more liquid-like viscoelastic response. At higher frequencies, an increase in both moduli is observed, with a change in the material behaviour. From about 0.3 Hz, G’ was found to be higher than G’’, suggesting a more solid-like response. This behaviour can be attributed to short-time scale chain dynamics and faster relaxation mechanisms. The modest frequency dependence of the moduli suggested a stable network with long relaxation times, typical of gel-like polymeric systems. Conversely, rHA displayed significantly lower moduli, with G′ consistently exceeding G″ over the entire frequency range, indicating a predominantly elastic response despite low absolute moduli.

Differences in rheological behavior related to HA molecular weight and source have been previously documented: hyaluronan preparations from bacterial versus animal sources and of different molecular weights show substantial variation in viscoelastic parameters [47], and zero-shear viscosity, complex viscosity, and crossover behavior have been shown to correlate directly with molecular weight [48]. Notably, the molecular weight and polydispersity of HA produced by recombinant microbial hosts are strongly dependent on the specific expression platform and strain, with several recombinant systems reported to yield lower and/or more polydisperse HA compared to conventional streptococcal fermentation [49,50], supporting the interpretation that the observed differences reflect production-platform-dependent molecular characteristics rather than an intrinsic effect of the recombinant origin.

These results demonstrated that, under the tested conditions, cHA_1 forms a more structured and mechanically stable network, whereas rHA displays a weaker elastic response and reduced network connectivity. Such differences are likely related to variations in polymer molecular characteristics, including molecular-weight distribution, chain entanglement density, and polydispersity, rather than to the recombinant origin itself.

### 2.7. Biocompatibility Evaluation

**Figure 7:**
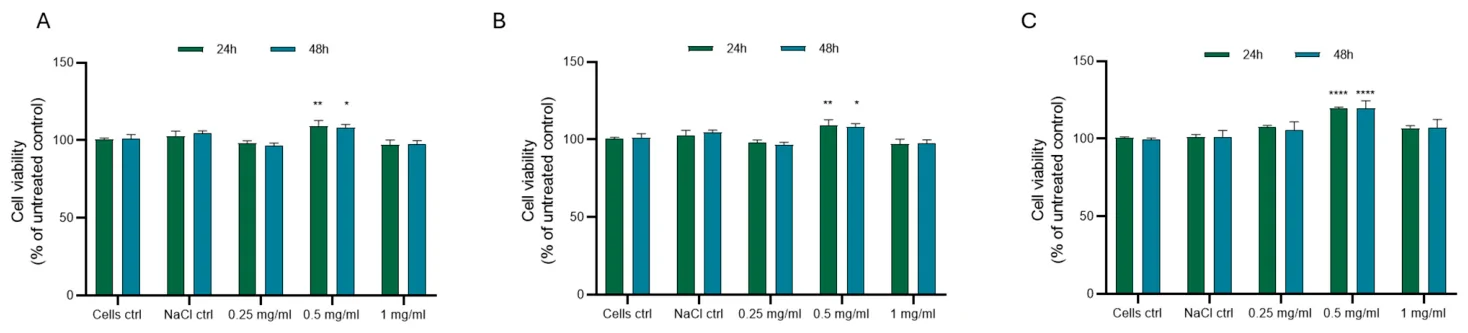
Cell viability on C2C12 cells treated with different concentrations of cHA_1 (**A**), cHA_2 (**B**) and rHA (**C**) for 24 and 48 h. Data are expressed as percentage of the untreated control and are reported as mean ± SD from three independent experiments, each performed with three technical replicates. Statistical analysis was performed by two-way ANOVA followed by Tukey’s multiple-comparisons test. Statistical significance between groups is indicated as * p < 0.05, ** p < 0.01 and **** *p* < 0.0001. Asterisks indicate comparisons with the untreated control.

The biocompatibility of rHA was evaluated in comparison with cHA_1 and cHA_2 at different concentrations (0.25 mg/mL, 0.5 mg/mL and 1 mg/mL) and exposure times (0, 24 and 48 h) using the SRB assay (Figure 7). Under all tested conditions, the relative SRB signal remained above 90% of the untreated control level, indicating no detectable reduction in cellular biomass (Figure 7).

A concentration-dependent increase in the relative SRB signal was observed for all formulations, with the strongest effect detected at 0.5 mg/mL. At 24 h, cHA_1 (Figure 7A), cHA_2 and rHA (Figure 7C) at 0.5 mg/mL significantly increased the relative SRB signal compared with untreated cells. A similar trend was observed at 48 h, although no difference was seen for either formulation at 1 mg/mL.

At 0.5 mg/mL, rHA showed numerically higher mean SRB signals than cHA_1 and cHA_2; however, these differences were not statistically significant at either 24 or 48 h (adjusted *p* > 0.05).

These findings indicate that rHA showed a cytocompatibility profile comparable to that of cHA_1 and cHA_2 under the tested conditions. The higher SRB signal observed at 0.5 mg/mL after 48 h may reflect changes in cellular biomass or protein content; however, the SRB assay alone does not distinguish between changes in cell number, cellular protein content, or cell attachment.

### 2.8. Scratch Assay

**Figure 8:**
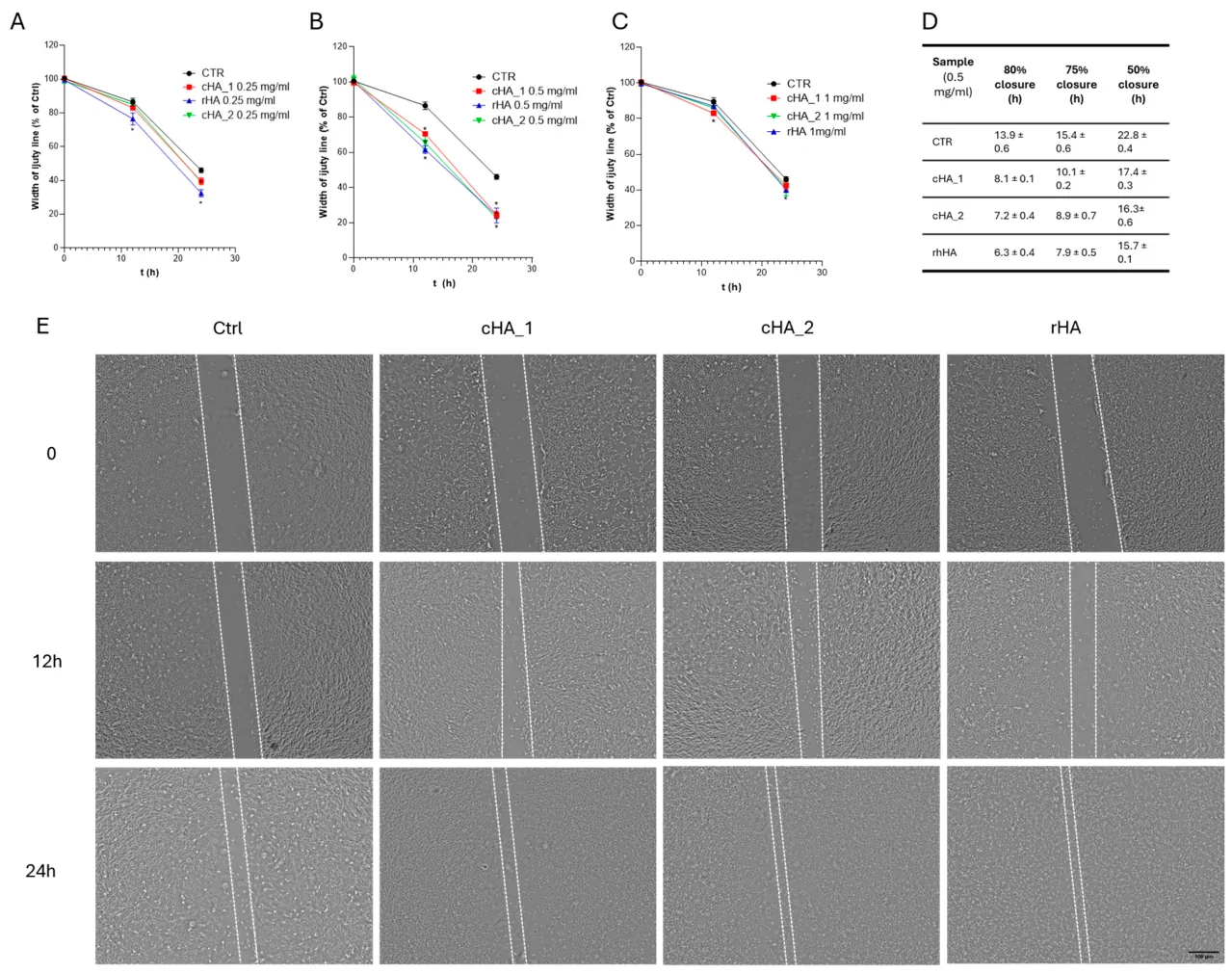
Effects of rHA and cHA on C2C12 cell wound closure. Representative time-course curves showing wound closure kinetics over 24 h for control (CTR, black line), cHA_1 (red line), cHA_2 (green line) and rHA (blue line) at 0.25 mg/mL (**A**), 0.5 mg/mL (**B**) and 1 mg/mL (**C**). Summary table showing time to achieve 50%, 75%, and 80% wound closure for each sample, calculated by linear interpolation (**D**). Data are expressed as mean ± SD of three independent experiments performed in triplicate. Statistical analysis was performed by two-way ANOVA. Representative optical microscopy images of C2C12 cell monolayers acquired using a 20× objective, showing wound progression over time (**E**). Images were converted to grayscale and white-balanced in post-processing to enhance cell contrast. FFT bandpass filtering was applied in addition to grayscale conversion. The scale bar shown in the bottom-right image represents 100 µm and applies to all images in panel E.

To further investigate the effects of HA on wound-closure dynamics, a scratch assay was performed. Preliminary time-course experiments were conducted at different HA concentrations (0.25, 0.5, and 1 mg/mL) and time points (12 and 24 h) (Figure 8, panels A–C).

Based on these data, 0.5 mg/mL was selected for comparative analysis, as it consistently induced the highest wound closure rate. At 24 h, treatment with rHA at 0.5 mg/mL significantly enhanced wound closure compared to untreated controls (Figure 8B,D,E). No statistically significant differences were observed between rHA (Figure 8B) and both used cHA (Figure 8B) (*p* > 0.05), indicating that rHA and both cHA displayed comparable wound-closure activity under the tested conditions (Figure 8B,E).

Taken together, these findings suggest that rHA may promote cell migration, a crucial process in tissue repair and regeneration, without showing statistically significant differences from cHA_1 and cHA_2 under the tested conditions (*p* > 0.05). This effect is consistent with HA’s known role in modulating cell adhesion, motility and extracellular-matrix interactions, although the present assay does not identify the underlying receptor-mediated mechanisms.

## 3. Discussion

The rHA produced via fermentation of *E. coli* and purified through TCA treatment and activated carbon adsorption was obtained as a white, fibrous powder. Protein analysis confirmed the effectiveness of the purification strategy, with levels decreasing from 7.47 mg/L in unpurified HA to 0.48 mg/L in the purified product (Figure 1). The combined UV spectrophotometric and biochemical analyses confirmed the effectiveness of the purification strategy applied to rHA. The reduction in absorbance at 280 nm in purified samples suggests a marked decrease in protein-related contaminants, consistent with the results obtained from the Bradford assay. Similarly, the decrease in A260 values indicates a reduction in nucleic-acid-associated absorbance in the purified rHA samples [29]. Electrophoretic analyses supported the specificity of the purification, showing sharper and better-defined HA-compatible bands without detectable CS-like staining (Figure 2).

It is noteworthy that HA electrophoretic profiles should be interpreted with consideration of the separation conditions and sample composition. Buffer composition can influence HA separation in agarose gels, while association with HA-binding proteins can alter electrophoretic mobility and interfere with molecular mass estimation [37]. Enzymatic digestion with hyaluronidase further supported the identity of the purified rHA as an HA-like polymer susceptible to canonical HA enzymatic cleavage (Figure S1).

The differential staining behavior of chondroitin sulphate with Stains-All, together with its resistance to hyaluronidase digestion, further validated the substrate specificity of the enzymatic assay and provided no evidence of sulfated glycosaminoglycan contaminants in the rHA preparation.

These purification outcomes align with strategies reported in the literature for the purification of HA produced by bacterial fermentation, aimed at reducing protein- and nucleic-acid-associated contaminants prior to biocompatibility assessment. In particular, adsorption-based purification using activated carbon has been reported to reduce endotoxin and high-molecular-weight protein contaminants in HA produced from Streptococcus cultures, yielding a highly biocompatible product with low residual impurities [51]. Also endotoxin levels were effectively reduced following purification.

However, because recombinant HA intended for biomedical translation must comply with strict purity and safety requirements, future investigations should include direct quantification of residual host–cell DNA, as well as a more comprehensive assessment of process-related impurities.

The structural characterization by FT-IR spectroscopy revealed that rHA retained the characteristic absorption bands of natural HA, including hydroxyl, amide, carboxylate, and saccharide groups (Figure 3). Minor variations in peak intensity are likely associated with a different molecular weight distribution or production method. These results are in agreement with documented polydispersity of HA produced via microbial systems, where fermentation products typically show a broad molecular weight range depending on host strain and cultivation parameters [52].

Further characterization by DLS revealed an apparent hydrodynamic diameter of 495 nm and a polydispersity index (PdI) of 0.427 for rHA. The relatively high PdI indicates a heterogeneous size distribution in aqueous solution, suggesting that the measured hydrodynamic diameter may be influenced by the presence of larger assemblies or aggregates. Given the flexible, polymeric nature of HA, its hydrodynamic behavior may also depend on chain conformation and intermolecular interactions in solution. Therefore, the measured diameter should not be interpreted as a direct measure of individual HA chain dimensions or molecular weight. Rather, these findings provide information on the apparent hydrodynamic properties of rHA under the experimental conditions used, while highlighting the limitations of DLS in characterizing heterogeneous polymeric systems. Complementary analytical approaches are thus required to obtain a more comprehensive understanding of the physicochemical properties of rHA.

Molecular-size assessment was performed using validated HA gel electrophoresis methods. These approaches converged in indicating a polydisperse rHA preparation with low-to-medium molecular-size populations; mass photometry identified a dominant modal population around 194 kDa and a minor broader population higher than 500 kDa (Supplementary Figure S2).

According to the molecular-weight classification [5,11], rHA thus represents a heterogeneous preparation enriched in low-to-medium molecular-size populations, a profile that is consistent with its inferior viscoelastic moduli relative to the high-molecular-weight commercial references.

However, because gel electrophoresis provides migration-based apparent molecular-mass estimates and mass photometry provides modal native-solution mass estimates within a calibration-dependent framework, future studies should include chromatographic analyses to refine the molecular-weight distribution, dispersity and hydrodynamic behaviour of the recombinant polymer.

The present findings also underscore the importance of strict control of electrophoretic conditions, particularly buffer composition and protein removal, to ensure reliable interpretation of HA size distributions.

Rheological characterization indicated the typical behaviour of a viscoelastic HA solution and confirmed shear-thinning under flow. In these experiments, cHA_1 behaved as a more structured, gel-like polymer network, while rHA showed a lower-viscosity and more processable viscoelastic profile (Figure 6). From an application perspective, these results suggest that the tested cHA could provide higher structural stability, whereas rHA may offer enhanced processability and easier handling under shear conditions. These differences are consistent with, but do not by themselves prove, the molecular-size distribution and reduced chain entanglement inferred from the combined electrophoretic and mass photometry profiles. Importantly, these differences should not be interpreted as intrinsic effects of recombinant versus commercial origin, as molecular-weight distribution and polydispersity are major determinants of HA rheological behaviour.

TGA demonstrated that initial weight loss (30–150 °C) corresponded to water evaporation, consistent with HA’s hygroscopic nature (Figure 5). Major degradation occurred between 150–250 °C, due to glycosidic bond cleavage, followed by gradual combustion of residual material above 300 °C. The thermal profile is comparable to that reported for natural and microbially derived HA, which also show stepwise water loss followed by main polymer backbone degradation in similar temperature ranges [46]. The earlier onset of polymer decomposition in rHA (≈150 °C vs. ≈250 °C for cHA_1) may reflect a lower average molecular weight and reduced chain entanglement, consistent with the polydisperse molecular-size profile indicated by gel electrophoresis, rather than a fundamental structural difference from native HA.

SRB assay results indicated that rHA did not induce detectable cytotoxicity in C2C12 cells at concentrations up to 1 mg/mL under the conditions tested (Figure 7). Treatment with rHA at 0.5 mg/mL was associated with a modest increase in SRB signal, which may reflect increased cellular biomass; however, this finding should not be interpreted as direct evidence of enhanced cell proliferation and was also evident with cHA.

In the scratch assay, rHA treatment significantly increased the rate of wound closure compared with controls (Figure 8). This finding indicates an enhanced closure response in the C2C12 monolayer under the experimental conditions tested; however, because scratch closure can reflect both cell migration and proliferation, the results do not allow these mechanisms to be distinguished. Accordingly, the observed effect should be interpreted as a preliminary indication of a potential influence of rHA on cellular wound-closure dynamics. Further studies using proliferation-specific assays and migration assays performed under conditions that minimize cell proliferation, together with mechanistic analyses, will be required to determine whether the observed effects are primarily attributable to altered proliferation, migration, or both.

The biological findings should be interpreted in the context of the specific rHA, cHA_1, and cHA_2 preparations investigated in this study. HA biological activity is strongly dependent on molecular size, and differences in molecular-size distribution may therefore contribute to the cellular responses observed here. Although cHA_2 showed partial overlap with rHA in the apparent molecular-mass range detected, their distribution profiles differed; consequently, cHA_2 should not be regarded as a molecular-weight-matched comparator. The present experiments therefore do not establish general biological equivalence between recombinant and commercial HA, nor do they allow the observed cellular effects to be attributed specifically to the recombinant origin of the polymer. Comparisons using preparations with matched molecular-size distributions and comparable purity would be required to assess effects associated with production origin.

Further studies using molecular-weight-matched HA preparations with comparable molecular-size distributions would be required to directly assess the intrinsic biological properties of the two materials.

Taken together, the physicochemical and biological data consistently suggest that rHA represents a structurally authentic but molecularly distinct HA preparation compared to the commercial references tested. Its lower apparent molecular size and reduced chain entanglement, while determining inferior viscoelastic moduli, may confer specific advantages in formulation contexts where fluidity, filterability or cell–matrix accessibility are prioritized over gel-forming capacity. These considerations further underscore the importance of molecular-size-matched comparisons in future studies aimed at disentangling production-origin effects from molecular-weight-dependent biological responses.

In conclusion, the produced rHA showed markedly reduced protein content and nucleic-acid-associated absorbance after purification, together with preserved chemical identity, hyaluronidase susceptibility, and favourable preliminary biological profile characterized by the absence of detectable cytotoxicity under the conditions tested and an increased scratch-closure response in C2C12 cells, while exhibiting physicochemical and rheological properties distinct from those of commercial HA. These differences are compatible with a polydisperse apparent molecular-size distribution and reduced chain entanglement, which may confer enhanced fluidity and processability. The addition of mass photometry strengthens the molecular-size characterization by providing an orthogonal solution-based estimate, but the reported values should be interpreted as approximate modal masses rather than absolute molar-mass averages. Absolute Mw, Mn and dispersity will still require future SEC-MALS, SEC-TDA, GPC-MALS or an equivalent validated chromatographic/light-scattering characterization.

Despite being limited to in vitro characterization and to molecular-size assessment by gel electrophoresis plus mass photometry, this study provides a solid foundation for further investigations into the functional performance and potential applications of recombinant HA.

## 4. Materials and Methods

### 4.1. Materials

Tris–borate (cod. T1503; 308093), EDTA (cod. 5134), bromophenol blue (cod. 114391), sucrose (cod. S0389), Stains-All (cod. 230960), chondroitin sulphate (CS, cod. H5388), hyaluronidase (cod. H1136), and cHA_1 (cod. 53747) were purchased from Merck (Darmstadt, Germany).

The cHA_1 was supplied as hyaluronic acid sodium salt from *Streptococcus equi*, with a manufacturer-reported molecular weight of approximately 1.5–1.8 MDa and protein impurities ≤1%. HA standard mixture was purchased from Echelon Bioscience (Salt Lake City, UT, USA); cHA_2 (cod. 7W-GC0118) was purchased from Cymitquimica (Barcelona, Spain) (molecular weight 200–400 kDa).

If not otherwise stated, other analytical grade chemical reagents were purchased from Merck (Darmstadt, Germany).

### 4.2. HA Production: Fermentation, Extraction and Purification

The HA used in this study was produced by microbial fermentation in *E. coli* using a proprietary recombinant system. Due to ongoing intellectual property protection, detailed information regarding strain engineering, fermentation conditions, and production yields cannot be disclosed. Although proprietary upstream details cannot be disclosed, all downstream purification and analytical characterization procedures are described in sufficient detail to allow independent evaluation of the purified material.

Following production, HA was recovered and subjected to purification steps aimed at removing cellular components, proteins, and other impurities, yielding a well-purified polymer suitable for subsequent analyses.

The extracted rHA was purified according to a procedure adapted from Karami et al., 2021 [40], with minor modifications. In brief, the crude extract was first treated with 1% activated carbon to adsorb residual proteins, nucleic acids and impurities. The carbon was subsequently removed by centrifugation (7000 rpm for 20 min at room temperature). Next, one volume of 100% trichloroacetic acid (TCA) was added to ten volumes of the rHA-containing supernatant to precipitate residual acid-insoluble and other impurities. After centrifugation at 7000 rpm for 10 min, the precipitated impurity-containing pellet was discarded, whereas the HA-containing supernatant was carefully recovered for further processing. The pH of the recovered supernatant was adjusted to approximately 7.0. The neutralized HA-containing solution was subsequently diluted fivefold with double-distilled water, filtered through a 0.45 µm membrane filter (Merck Millipore, SLCRM25NS), freeze-dried, and stored at 4 °C.

### 4.3. Yield Evaluation

The yield of rHA was measured using the carbazole method [53] with absorbance recorded at 530 nm. Briefly, 40 μL of aqueous HA containing sample was mixed with 200 μL of chilled sulfuric acid containing 0.025 M sodium tetraborate, keeping the mixture on ice. After mixing, the tubes were placed in a boiling water bath for 10 min, then immediately cooled in an ice bath. 8 μL of 0.25% carbazole in ethanol were added, thoroughly mixed, and the reaction was heated again in boiling water for 10 min, followed by cooling on ice. Samples containing hyaluronic acid developed a characteristic purple colour. Absorbance was measured at 530 nm. Quantification was performed by comparison to a standard curve prepared from cHA_1 at 0.25 mg/mL, 0.5 mg/mL, 1 mg/mL and 2.5 mg/mL.

### 4.4. Purity Assessment

Protein concentration was determined using the Bradford assay [54,55] in rHA resuspended at 1% in distilled water. A 5:1 ratio of Bradford reagent to sample was prepared, and the mixture was incubated for 5 min at room temperature with continuous mixing. Absorbance was then recorded at 595 nm, and protein content was quantified based on a calibration curve. Similarly, the concentration of nucleic acids was estimated by measuring the absorbance at 260 nm using a NanoDrop spectrophotometer 2000 (Thermo Scientific) [NanoDrop 2000/2000c, SpectrophotometerV1.0, Thermo Scientific, Wilmington, DE, USA, 2009].

### 4.5. UV-Visible Spectrum

All the tested HA suspensions were prepared at a final concentration of 0.2 mg/mL in double-distilled water (ddH_2_O), as previously described by Choi et al. 2010 [56]. UV–Visible spectroscopic analysis of the HA solution was carried out at 25 °C using a Beckman DU800 spectrophotometer. Absorbance spectra were recorded over a wavelength range of 205–450 nm to capture the characteristic absorption features of HA. All measurements were performed in quartz cuvettes with a 1 cm optical path length. Each sample was analysed in triplicate to ensure reproducibility.

### 4.6. LAL-Assay

Endotoxin levels were quantified using the Pierce™ Chromogenic Endotoxin Quant Kit (Thermo Fisher Scientific), according to the manufacturer’s instructions [57]. Briefly, the lyophilized Escherichia coli endotoxin standard was reconstituted with endotoxin-free water and serially diluted to prepare a standard curve in the range of 0.01–0.1 EU/mL. Samples were appropriately diluted in endotoxin-free water (3 mg/mL) and adjusted to pH 6–8 when necessary. All standards and samples were analyzed in triplicate, together with a reagent blank.

For the assay, 50 μL of each standard or sample was added to a 96-well plate, followed by 50 μL of reconstituted Limulus Amebocyte Lysate (LAL) reagent. The plate was incubated at 37 °C for the time specified by the manufacturer, after which 100 μL of chromogenic substrate was added to each well. Following a further incubation at 37 °C for 6 min, the reaction was stopped by adding 50 μL of 25% acetic acid. Absorbance was measured at 405 nm using a microplate reader. The mean absorbance of the blank was subtracted from the absorbance of the standards and samples. Endotoxin concentrations were calculated from a linear regression of the blank-corrected absorbance values of the standards versus their corresponding endotoxin concentrations (EU/mL). Only standard curves with a coefficient of determination (R^2^) ≥ 0.98 were accepted.

Results were then normalized to the HA concentration of the corresponding sample and expressed as endotoxin units per milligram of HA (EU/mg HA).

### 4.7. Gel Electrophoresis

The apparent molecular mass distribution of HA samples was estimated by gel electrophoresis, following the method of Bhilocha et al. (2011) [37], with interpretation restricted to migration-based, semi-quantitative comparison against HA molecular weight standards. Polyacrylamide gels (4–20%, 10.1 × 7.3 cm) and 2% agarose gels (7 × 10 cm) were prepared in 1X Tris–borate EDTA (TBE) buffer (100 mM Tris–borate and 1 mM EDTA, pH 8.3). HA samples (both rHA and cHA) were prepared at a concentration of 0.5 µg/µL in double-distilled water. A 5 µL aliquot of each HA sample was mixed with 10 µL of water and 3 µL of loading buffer (0.02% bromophenol blue and 2 M sucrose in 1X TBE).

Particular attention was paid to buffer composition and sample purity, as HA electrophoretic mobility is highly sensitive to ionic conditions and the presence of residual proteins, which may induce migration artefacts or anomalous band focusing. All buffers were therefore freshly prepared in TBE and pre-equilibrated to minimize variability [37].

TBE-polyacrylamide gel electrophoresis was performed at room temperature at a constant voltage of 200 V for 1 h, whereas agarose electrophoresis was performed at 20 V for 30 min followed by 40 V for 3.5 h. After electrophoresis, gels were stained in approximately 500 mL of 0.005% Stains-All in 50% ethanol overnight under light-protected conditions at room temperature. The gels were then transferred into 10% ethanol destaining solution and stored in the dark for 1 day, with at least one replacement of the destaining solution. Final removal of residual background staining was achieved by placing the gel on a light box for a few minutes. Molecular size estimates were obtained by comparison with HA standards and should be interpreted as apparent molecular mass values rather than absolute hydrodynamic molecular weight parameters. Gel images were acquired under fixed distance and exposure conditions, without automatic image processing.

Electrophoretic separation of HA samples was analysed by densitometry using Fiji/ImageJ software (version 1.54p) on Windows 64-bit. Gel images were converted to 8-bit grayscale prior to analysis. Individual lanes were defined using identical rectangular ROIs, and densitometric profiles were obtained as gray value intensity versus migration distance (pixel position). For polyacrylamide gels, background subtraction was performed using the rolling ball algorithm with a radius of 50 pixels. For agarose gels, a vertical background intensity profile was extracted from a region adjacent to each lane, using the same vertical position and height as the corresponding lane ROI. This background profile was subtracted point by point from the lane intensity profile at each migration position.

HA molecular weight standards were electrophoresed in parallel with unknown samples to generate a calibration curve. Migration distances of the HA standards were measured from the top of the gel, and a calibration curve was generated by linear regression analysis of log molecular weight versus migration distance (pixel position). Only standards within the linear separation range of the gel were included in the regression analysis (R^2^ > 0.98).

The limits of the plateau reported for the PAGE profile were defined as the migration positions at which the normalized intensity fell below 90% of the maximum value.

The calibration equation derived from the HA standards was subsequently used to convert migration distance values into apparent molecular weight distributions for each sample. Due to the non-globular and highly hydrated nature of HA, migration-based molecular weight estimates reflect apparent rather than absolute molecular sizes.

Densitometric profiles on PAGE were normalized to the total area under the curve to allow comparison of HA molecular weight distribution profiles among samples. Normalization was performed after background subtraction to the total integrated density of each lane.

For agarose gels, each background-corrected densitometric profile was normalized by dividing all intensity values by the maximum corrected intensity of that lane over the entire analyzed migration interval. This procedure set the maximum intensity of each profile to 1, allowing comparison of profile shapes but not relative HA abundance between samples.

Linear regression analyses and graphical representations were generated using GraphPad Prism (GraphPad Software 8.0.2., San Diego, CA, USA).

### 4.8. Fourier Transform Infrared Spectroscopy

The molecular fingerprint of the samples was analysed by Fourier transform infrared (FT-IR) spectroscopy. HA samples (both rHA and cHA) were resuspended in ddH_2_O at a concentration of 0.5 mg/mL, deposited onto a germanium window, and allowed to dry under a fume hood for 24 h under controlled ambient conditions. FT-IR spectra were acquired over the 4000–400 cm^−1^ range using an FTIR-6300 spectrometer (Jasco GmbH, Pfungstadt, Germany). All measurements were performed under identical acquisition settings to ensure comparability between samples.

### 4.9. Particles Size

DLS analysis was conducted using a Malvern Zetasizer Nano ZS 90 (Worcestershire, UK). An evaluation of hydrodynamic diameter was made at 25 °C by measuring the autocorrelation function at a 90° scattering angle. Samples of rHA and cHA resuspended in water at a final concentration of 0.1% were sonicated for 1 min by using a Sonorex RK 102H ultrasonic water bath (Bandelin Electronic, Berlin, Germany, www.bandelin.com (accessed on 1 October 2026)) before analysis.

### 4.10. Thermal Analysis

The thermal stability of rHA was evaluated by thermogravimetric analysis (TGA) using an SDT Q600 instrument (TA Instruments, New Castle, DE, USA). Samples (approximately 5–10 mg) were accurately weighed and placed in alumina crucibles prior to analysis. The system was equilibrated at 25 °C for 5 min to ensure thermal stabilization, after which samples were heated from 25 °C to 800 °C at a constant rate of 5 °C/min.

All measurements were performed under a continuous nitrogen flow of 50 mL/min to provide an inert atmosphere and prevent oxidative degradation, using an empty alumina crucible as a reference. The weight loss as a function of temperature was recorded, and thermograms were analysed to assess the thermal degradation profile of rHA. Each experiment was carried out in duplicate to ensure reproducibility.

### 4.11. Rheological Properties

HA is a natural polymer with intrinsic viscoelastic properties. HA suspensions (both rHA and cHA) were prepared by dispersing the polymer in double-distilled water to obtain a final concentration of 5% (*w*/*v*). Samples were gently mixed until complete dispersion and subsequently analysed as described below.

Rheological measurements were performed using a Kinexus Pro rotational rheometer (NETZSCH Pumpen & Systeme GmbH, Waldkraiburg, Germany) equipped with a parallel-plate geometry. A few millilitres of each suspension were carefully loaded onto the measuring system, and the gap distance was set at 2 mm. The temperature was maintained at 25 °C throughout the analyses, and samples were allowed to equilibrate for a short time before each measurement to ensure homogeneous temperature distribution.

Steady-state shear tests were initially carried out to evaluate the viscosity profile as a function of shear rate, according to the relationship:

η = τ/γ,(1)

where η is the dynamic viscosity, τ is the shear stress, and γ is the shear rate.

Oscillatory measurements were subsequently performed to investigate the viscoelastic properties of the suspensions. Strain sweep analyses were conducted at a constant frequency of 1 Hz over the strain range 0.1–100% to identify the linear viscoelastic region (LVR). Frequency sweep tests were then performed within the LVR over the frequency range 0.01–100 Hz at a fixed strain of 1%, allowing the determination of the storage modulus (G′) and loss modulus (G″).

All measurements were performed in triplicate under the same experimental conditions to assess the reproducibility of the analyses.

### 4.12. Cell Cultures

C2C12 murine myoblasts were cultured in sterile flasks (Corning, Corning, NY, USA) and maintained at 37 °C in a humidified 5% CO_2_ incubator (Forma Scientific, Waltham, MA, USA). Cells were grown in high-glucose DMEM (Sigma-Aldrich, St. Louis, MO, USA) supplemented with 10% FBS, 1% L-glutamine, 1% penicillin (100 U/mL), and 1% streptomycin (100 µg/mL). Cultures were handled under a laminar flow hood, and the medium was changed every two days. Cells were monitored by inverted microscopy and passaged at ~80–90% confluence. After aspiration of the medium, cells were washed with PBS (Ca^2+^/Mg^2+^-free) and detached with 0.05% trypsin/0.025% EDTA. Trypsin was neutralized with complete medium, and the cell suspension was centrifuged at 1000× *g* for 5 min at 25 °C (Beckman Allegra 6R). The pellet was resuspended in fresh medium and seeded into new flasks [58].

### 4.13. Biocompatibility Evaluation

The rHA biocompatibility against a cHA was investigated by means of the sulforhodamine B (SRB) assay [59]. Briefly, 100 μL of cell suspension (1 × 10^5^ cells/mL) were seeded into each well of a 96-well plate and incubated overnight to allow cell attachment. Subsequently, rHA and cHA, resuspended in 0.15 M NaCl, were added to the wells at final concentrations of 0.25, 0.5, and 1 mg/mL.

At predetermined time points, 100 μL of cold 10% (*v*/*v*) TCA were added to each well and incubated at room temperature for 30 min to fix cellular proteins. The plates were then washed five times with distilled water for 10 min per wash and air-dried overnight under a laminar flow hood.

Next, 70 μL of 0.4% (*w*/*v*) sulforhodamine B (SRB; Sigma-Aldrich 230162) in 1% (*v*/*v*) acetic acid (Sigma-Aldrich 45754) were added to each well and incubated at room temperature for 20 min. Excess dye was removed, and the plates were washed four times with 1% acetic acid to eliminate unbound stain.

The protein-bound dye was subsequently solubilized by adding 200 μL of 10 mM unbuffered Tris-base solution to each well, followed by incubation for 15 min on a rotary shaker at room temperature. Absorbance was measured at 492 nm using an Envision^®^ Multimode Plate Reader (PerkinElmer Inc., Waltham, MA, USA).

SRB assays were performed in three independent experiments with three technical replicates each (n = 9).

### 4.14. Scratch Assay

The ability of rHA to promote closure of a simulated wound was compared with that of cHA using a scratch assay. Briefly, C2C12 cells were seeded in 24-well plates at a density of 1.5 × 10^5^ cells/well. Once confluence was reached, 1 mm wide incision was made on the cell monolayer using a sterile pipette tip to simulate the wound. The progression of cell migration was monitored by means of an Optical Microscope (Leica Microsystems CMS) on snapshots taken immediately after the scraping and after at 12 h and 24 h. All snapshots were acquired with a 20X magnification. Cell migration measurements were carried out by quantifying the reduction in the distance between the edges of the wound during time in order to calculate the wound closure rate % [60]. The software Fiji/ImageJ (National Institute of Health, Bethesda, MD, USA) was used for the analysis [61]. For visual presentation in Figure 8E, raw micrographs were converted to grayscale and subjected to a background uniformization and white-balancing routine using Fiji/ImageJ software (version 1.54p) on Windows 64-bit to enhance contrast and facilitate the observation of the cell migration front. Wound-closure quantification was performed on the unprocessed images. Each sample was tested 3 times.

### 4.15. Statistical Analysis

Statistical analyses were performed using GraphPad Prism 8.0.2 (GraphPad Software, San Diego, CA, USA). Data are presented as mean ± standard deviation (SD) from at least three independent measurements, unless otherwise stated. Comparisons among experimental groups were performed using one-way or two-way analysis of variance (ANOVA), as appropriate, followed by Tukey’s multiple comparisons test. Differences were considered statistically significant at *p* < 0.05 [62].

## 5. Conclusions

In conclusion, this study investigated the physicochemical, rheological, and preliminary biological properties of a recombinant HA produced in E. coli and purified by activated carbon adsorption and TCA treatment. Purification markedly reduced residual protein content, nucleic-acid-associated absorbance, and endotoxin levels while preserving HA recovery. FT-IR spectroscopy and hyaluronidase digestion confirmed the chemical identity and structural integrity of the polymer. Gel electrophoresis and mass photometry indicated a polydisperse apparent molecular-size distribution with a predominant population centred around 194 kDa. Thermogravimetric analysis revealed a thermal degradation profile compatible with native HA, and rheological characterization showed shear-thinning behaviour with lower viscosity and viscoelastic moduli compared to the used commercial HA, consistent with the lower apparent molecular size. In C2C12 cells, rHA showed no detectable cytotoxicity up to 1 mg/mL. Taken together, these results support rHA as a purified, HA-compatible material with distinctive rheological properties and preliminary in vitro cellular performance.

Future studies should address absolute molecular-weight determination by SEC-MALS or equivalent methods, molecular-size-matched comparisons with commercial HA, and extended in vitro and in vivo biological evaluation to fully assess the translational potential of this E. coli-derived recombinant platform.

## Data Availability

The original contributions presented in this study are included in the article/Supplementary Material. Further inquiries can be directed to the corresponding author(s).

## Supplementary Materials

The following supporting information can be downloaded at: https://www.mdpi.com/article/10.3390/ijms27219997/s1. Reference [63] is cited here.

## Abbreviations

The following abbreviations are used in this manuscript:

HA	Hyaluronic acid
rHA	Recombinant hyaluronic acid
cHA	Commercial hyaluronic acid
CS	Chondroitin sulphate
