# Supplementary material for: Recombinant Hyaluronic Acid Produced in Escherichia coli: Purification, Molecular-Size Profile and In Vitro Cellular Performance
Source: Int J Mol Sci. 2026 Nov 8;27(21):9997. doi: 10.3390/ijms27219997 (PMC13764106; doi:10.3390/ijms27219997)
Supplement: Supplementary file 1 [file ijms-27-09997-s001.zip › ijms-4504723-supplementary.pdf]

S.1 Supplementary data: HA enzymatic digestion

HA samples (both rHA and cHA_1) were digested according to the protocol described by Yuan et al. [63], using 0.25 U of hyaluronidase per 1 μg of HA in a final reaction volume of 10 μL. Reactions were incubated at 37 °C for 3 h, after which enzymatic activity was inactivated by heating at 100 °C for 5 min.

Subsequently, gel electrophoresis was performed as described in the section 4.7. After digestion, samples were mixed with loading buffer to a final volume of approximately 20 μL before loading into 4-20% gradient polyacrylamide gel wells, and electrophoresis was carried out at 200 V for 1 h. Gels were then stained and imaged as described in paragraph 4.7.

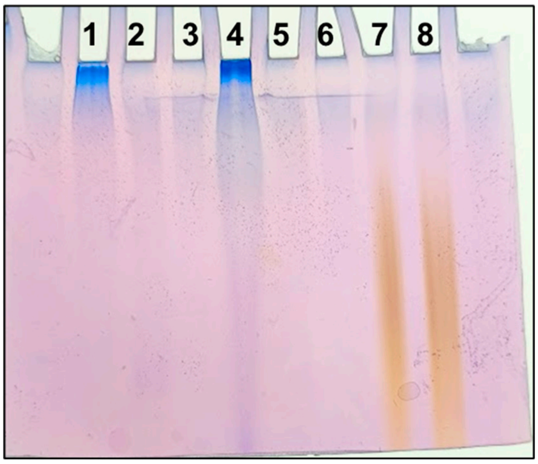

Figure S1. Enzymatic digestion of HA samples assessed by polyacrylamide gel electrophoresis and Stains-All staining. Lane 1: untreated cHA_1; Lane 2: hyaluronidase-digested cHA_1; Lane 3: empty; Lane 4: untreated purified rHA; Lane 5: hyaluronidase-digested rHA; Lane 6: empty; Lane 7: chondroitin sulphate (CS, negative control, untreated); Lane 8: hyaluronidase-digested CS. Blue staining indicates HA-positive species; yellow staining is characteristic of sulfated glycosaminoglycans (CS). The marked reduction in band intensity following hyaluronidase treatment (Lanes 2 and 5) confirms susceptibility of both cHA_1 and rHA to HA-specific enzymatic hydrolysis, supporting the HA identity of the recombinant polymer.

S.2 Supplementary data: mass photometry

Mass photometry was used as a complementary label-free method to estimate the native-solution mass distribution of cHA_2 and rHA. All measurements were recorded at 25°C using a Refeyn TwoMP mass photometer (Refeyn Ltd, UK). The instrument was calibrated with of a set of calibrants, β-amylase (56, 112, and 224 kDa), BSA (66kDa), and urease (90.7, 272, and 545 kDa). HA samples were resuspended in 10 mM Tris buffer at 0.5 mg/mL. For each measurement, a 2 μL aliquot of sample was applied to 8 μL of 10 mM Tris on a coverslip, resulting in a final concentration of approximately 100 μg/mL. Movies were acquired for 60 s using AcquireMP software (version R1) at 60 frames per second and a large field of view, and data were processed using DiscoverMP software (version R1.2). Mass estimation was performed by fitting Gaussian distributions to the mass histograms and reporting the modal value of each distribution. Each sample was analysed in triplicate. Because this analysis was introduced to complement gel electrophoresis rather than to derive chromatographic molar-mass averages,

mass photometry data are reported as approximate modal mass estimates and should not be interpreted as absolute Mw, Mn or dispersity values.

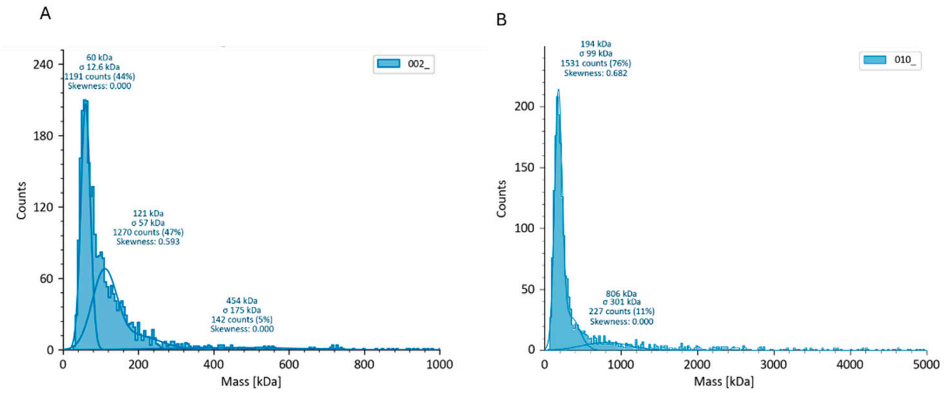

Figure S2. Mass photometry analysis of cHA_2 and purified rHA. Mass histogram showing the native-solution mass distribution of cHA_2 (A) and purified rHA (B)as determined by mass photometry at 25 °C.

The cHA_2 profile exhibited two major fitted populations centred at approximately 60 kDa (σ = 12.6 kDa; 1,191 events, 44%) and 121 kDa (σ = 57 kDa; 1,270 events, 47%), and a minor component centred at approximately 454 kDa (σ = 175 kDa; 142 events, 5%). In rHA a predominant population with an approximate modal mass of 194 kDa was detected (σ = 99 kDa, 1531 counts, 76% of detected events, skewness = 0.682). A less abundant higher-mass signal was also observed above the upper limit of the calibration range (509 kDa). These data support molecular heterogeneity and polydispersity of the rHA preparation but do not allow reliable assignment of an absolute mass value to the higher-mass fraction. Each measurement represents analysis of a single aliquot; three independent measurements were performed with comparable results. Mass photometry values should be interpreted as approximate native-solution modal masses rather than absolute molar-mass averages (Mw or Mn) or dispersity indices.
